# Impact of fundamental components of the Mediterranean diet on the microbiota composition in blood pressure regulation

**DOI:** 10.1186/s12967-024-05175-x

**Published:** 2024-05-03

**Authors:** Ana Karina Zambrano, Santiago Cadena-Ullauri, Viviana A. Ruiz-Pozo, Rafael Tamayo-Trujillo, Elius Paz-Cruz, Patricia Guevara-Ramírez, Evelyn Frias-Toral, Daniel Simancas-Racines

**Affiliations:** 1 Centro de Investigación Genética y Genómica, Facultad de Ciencias de la Salud Eugenio Espejo, Universidad UTE, Quito, 170527 Ecuador; 2 Escuela de Medicina, Universidad Espíritu Santo, Samborondón, 0901952 Ecuador; 3 Centro de Investigación de Salud Pública y Epidemiología Clínica (CISPEC), Universidad UTE, Quito, 170527 Ecuador

**Keywords:** Mediterranean diet, Microbiota, Blood pressure, Hypertension

## Abstract

**Background:**

The Mediterranean diet (MedDiet) is a widely studied dietary pattern reflecting the culinary traditions of Mediterranean regions. High adherence to MedDiet correlates with reduced blood pressure and lower cardiovascular disease (CVD) incidence and mortality. Furthermore, microbiota, influenced by diet, plays a crucial role in cardiovascular health, and dysbiosis in CVD patients suggests the possible beneficial effects of microbiota modulation on blood pressure. The MedDiet, rich in fiber and polyphenols, shapes a distinct microbiota, associated with higher biodiversity and positive health effects. The review aims to describe how various Mediterranean diet components impact gut microbiota, influencing blood pressure dynamics.

**Main body:**

The MedDiet promotes gut health and blood pressure regulation through its various components. For instance, whole grains promote a healthy gut microbiota given that they act as substrates leading to the production of short-chain fatty acids (SCFAs) that can modulate the immune response, preserve gut barrier integrity, and regulate energy metabolism. Other components of the MedDiet, including olive oil, fuits, vegetables, red wine, fish, and lean proteins, have also been associated with blood pressure and gut microbiota regulation.

**Conclusion:**

The MedDiet is a dietary approach that offers several health benefits in terms of cardiovascular disease management and its associated risk factors, including hypertension. Furthermore, the intake of MedDiet components promote a favorable gut microbiota environment, which, in turn, has been shown that aids in other physiological processes like blood pressure regulation.

## Background

The term “Mediterranean diet” (MedDiet) was first coined by Ancel Keys in 1960, and it has now become one of the most extensively studied and widely adopted dietary patterns worldwide [1]. Originating from the Mediterranean Sea, it predominantly reflects the dietary habits of Greece and various regions of Italy, Spain, and Morocco. Notably, the MedDiet has gained recognition as an intangible cultural heritage by the United Nations Educational, Scientific and Cultural Organization (UNESCO) [2, 3]. Reports have described a higher systolic and diastolic blood pressure in European countries (136/83 mmHg), in comparison with North America (127/77 mmHg) [4]. Interestingly, Greece, one of the countries in where the MedDiet was born, has blood pressure values similar to those reported in North America. However, hypertension rates have been increasing in the country as the population moves away from their MedDiet [5].

Traditionally, the dietary habits of Mediterranean countries emphasize the consumption of carbohydrates/whole grains, olive oil, fruits, vegetables, wine, fish, and lean proteins, while minimizing intake of red meat and processed foods [6, 7]. As a result, this diet is considered to be plant-based and semi-vegetarian [3].

MedDiet has gained attention due to its associated health benefits [8–16]. For instance, research has shown an inverse association between MedDiet adherence and the incidence and mortality of coronary heart disease and cardiovascular disease (CVD), along with a notable reduction in blood pressure [1, 17–21]. Elevated blood pressure is one of the principal risk factors linked to CVD, dementia, and chronic kidney disease [22]. Therefore, it is of paramount importance to control blood pressure, and one of the potential methods for this regulation is through diet [23, 24].

Moreover, the microbiota, which is immensely influenced by diet, plays a key crucial role in cardiovascular health. Under healthy conditions, it is primarily composed of Firmicutes, Bacteroidetes, Proteobacteria, and Actinobacteria. However, in individuals with CVD, the gut microbiota is dysregulated. For instance, various gut microbiome studies have shown that an increased abundance of Gram-negative bacteria, such as *Prevotella* and *Klebsiella*, has been correlated with higher blood pressure [25]. Microbial interactions within the gut occur through either antagonistic or symbiotic relationships [25]. Notably, research has shown that certain gut bacteria can use sulfate as a substrate, producing hydrogen sulfide (H_2_S), which in turn, has the capacity to induce blood vessel relaxation, thereby regulating blood pressure [26].

Hypertensive patients exhibit a decrease in microbial richness and diversity [27, 28]. These findings suggest that targeted modulation of the gut microbiota could have potential benefits in blood pressure regulation. Furthermore, the gut microbiota generates diverse metabolites, and an imbalance (dysbiosis) in the microbiota could further lead to blood pressure imbalances [29]. For instance, specific types of bacteria produce short-chain fatty acids (SCFA), and reports suggest that SCFA possesses regulatory effects on blood pressure via the G protein-coupled receptor 41 (GPR-41) and the Olfatory receptor 78 (Olfr78), as observed in model organisms [25]. GPR-41 modulates blood pressure by decreasing active vascular tone without modifying passive features of blood vessels [30]. Similarly, Olfr78 can regulate blood pressure by mediating renin secretion in response to SCFAs [31, 32]. Consequently, SCFA emerges as a potential therapeutic target for hypertension [25].

The MedDiet has been shown to have the capacity to modulate the human microbiota. In this dietary pattern, high amounts of fiber and polyphenols are ingested, increasing prebiotic action on specific strains. Additionally, this diet stimulates the increased production of SCFA, which is beneficial in blood pressure management [33]. Notably, the microbiota shaped by the MedDiet, which is associated with a higher microbiota biodiversity and positive effects on human health, differs significantly from that influenced by a Western-type dietary pattern. The Western diet is characterized by increased levels of Bacteroides, while the MedDiet promotes an increased proportion of the genus Prevotella [34, 35]. Furthermore, specific components of the MedDiet have been linked to the presence of particular strains in the gut microbiota. For example, the consumption of vegetables is related to a higher presence of Dorea, Ruminococcus, Alistipes, and Rikenellaceae [36].

The purpose of the present review is to elucidate and highlight the impact that various components of the MedDiet exert on gut microbiota and its effects on blood pressure.

## Methods

The information was compiled from different databases. The principal search engines used were Google Scholar and PubMed. The search terms were used individually and in combination, and these were: “Mediterranean diet”, “health benefits”, “microbiota”, “hypertension”, “blood pressure regulation”, “whole grains”, “extra virgin oil”, “fruits and vegetables”, “wine”, “fish and lean proteins”, “meat consumption”, and “harmful effects”. The selected articles included were primarily published in the last ten years; however, pivotal studies were included regardless of publication date.

## Mediterranean diet on gut microbiota composition, and blood pressure regulation

Traditionally, the dietary habits of Mediterranean countries emphasize the consumption of carbohydrates/whole grains, olive oil, fruits, vegetables, wine, fish, and lean proteins, while minimizing intake of red meat and processed foods [6, 7]. Beyond the cardiovascular benefits associated with the MedDiet [37], research suggests that it also play a role in shaping gut microbiota composition [38], which, in turn has been implicated in the development and pathogenesis of blood pressure [39].

Exploring the relationship between the MedDiet, gut microbiota and blood pressure is crucial for advancing our understanding of their interconnectedness and potential health benefits.

### Whole grains

Whole grains are a fundamental component of the MedDiet, and their consumption has been linked with numerous health benefits [40]. Rich in fiber, vitamins, minerals, and phytochemicals, whole grains provide a balanced mix of soluble and insoluble fiber [41, 42]. Several studies [41, 42] have demonstrated their protective effects against cardiovascular diseases, including hypertension [42–45]. This protective effect is attributed to the improvement of lipid profiles, reduction of inflammation, enhancement of endothelial function, and consequent regulation of blood pressure [44, 46].

Furthermore, the impact of whole grain consumption on gut microbiota composition has been extensively researched. Whole grains serve as sources of dietary fiber, indigestible by humans, which act as substrates for microbial fermentation in the gut, producing of short-chain fatty acids such as acetate, propionate, and butyrate [47, 48]. Dietary fibers, including soluble and insoluble varieties, play a crucial role in overall well-being. Soluble fibers can absorb water, resulting in delayed gastric emptying, prolonged food transit time, and reduced nutrient absorption. In contrast, insoluble fibers promote rapid gastric emptying and increased fecal bulk, thereby aiding in relieving constipation [49, 50].

While both types of fibers are indigestible and can be fermented by gut bacteria, soluble fibers are more readily fermented and serve as a source of SCFAs. These fatty acids, particularly propionic acid, have been shown to reduce cholesterol synthesis in the liver, leading to decreased blood cholesterol levels. Despite controversies regarding the roles of soluble and insoluble fibers, it is widely acknowledged that dietary fibers improve cardiovascular health [49, 50].

Moreover, SCFAs play critical roles in modulating the immune response, preserving gut barrier integrity, and regulating energy metabolism [51].

#### Butyrate

Changes in the abundance of specific bacteria can influence butyrate production. An increase of Firmicutes, such as *Roseburia, Eubacterium rectale, Faecalibacterium prausnitzii*, and many *Clostridium* species, leads to higher butyrate production [52]. Butyrate has been shown to maintain gut barrier integrity by increasing the production of integral proteins involved in the forming junctions between colonocytes [52]. Several studies have demonstrated a direct relationship between intestinal inflammation, gut epithelial barrier dysfunction, and elevated blood pressure levels [53–55]. For instance, it has been shown that butyrate can stabilize the hypoxia-inducible factor-1, promoting vascularization and reducing blood pressure [56]. Therefore, the abundance of butyrate-producing bacteria in the human gut is associated with lower blood pressure levels [55].

#### Acetate

Produced by gut microbiota during the fermentation of dietary fiber, acetate has been linked to various physiological effects. Research suggests that an increasing *Bifidobacterium*, *Ruminococcus bromii* and *Akkermansia muciniphila* is associated with higher acetate production [52, 57]. Elevated hypothalamic acetate levels have been shown to increase oxidative lactate production and neurotransmission [52]. The increase in acetate-producing bacteria has been correlated with beneficial effects on blood pressure regulation [58]. Conversely, a decrease in these bacteria due to dysbiosis has been linked to cardiovascular disorders [59].

#### Propionate

Influenced by specific bacterial taxa associated with whole grain consumption, propionate is primarily produced by bacteria belonging to the Bacteroidetes phylum [60]. An increase in propionate-producing bacteria has been linked to improvements in beta-cell function and insulin resistance, suggesting a potential role in blood pressure control [61]. Additionally, a recent study found that propionate decreased blood pressure, inflammation, and atherosclerosis, while improving the vascular dysfunction via reduction in T_H_17 cells [62]. Therefore, an increased in propionate-producing bacteria suggest a potential role in blood pressure control.

Furthermore, the gut microbiota has been implicated in mediating the effects of whole grain consumption on blood pressure regulation, while dysbiosis has been associated with hypertension [59]. Manipulation of the gut microbiota through dietary interventions such as whole grain consumption could mitigate hypertension, thereby improving cardiovascular outcomes.

### Extra virgin olive oil

Olive oil is a cornerstone of the MedDiet, renowned for its richness in tocopherols, carotenoids, and polyphenols, all of which harbor antioxidant and anti-inflammatory properties beneficial for cardiovascular health [63–65]. The MedDiet, enriched with olive oil, may modulate the abundance of specific bacterial taxa associated with cardiovascular health [66].

Among the polyphenols found in olive oil, hydroxytyrosol stands out for its anti-inflammatory and anti-teratogenic activity, contributing to improving of lipid profiles and reduction of oxidative stress [67, 68]. Furthermore, polyphenols in olive oil may influence gut microbiota composition, thereby impacting blood pressure regulation [69]. Consequently, the MedDiet, emphasizing on olive oil consumption, has been linked to reduced systemic inflammation and improved vascular function, pivotal factors in maintaining healthy blood pressure levels [70, 71].

Recent investigations have also explored the potential mechanisms linking olive oil consumption, gut microbiota composition, and blood pressure regulation. It has been observed that olive oil impacts the microbiota, with consumption associated with increased abundance of *Lactobacillus, Bifidobacterium*, and *Clostridium* in the gut microbiota [68, 72, 73]. Specifically, *Clostridium cocleatum*, in the presence of *Lactobacillus* undergoes fermentation acting as a probiotic. Furthermore, *Lactobacillus* and *Bifidobacterium* acts as prebiotic due to their antioxidant activity [68]. The presence of polyphenol is associated with the presence of *Bacteroides*, which are linked to a protective effect reducing the blood pressure [68, 74, 75].

The consumption of olive oil as a central component of the MedDiet, has been correlated with favorable effects on gut microbiota composition, resulting in a blood pressure regulation [68, 72]. Emerging evidence suggests that olive oil-derived bioactive compounds modulate vascular function and inflammation, potentially mediated by alterations in the gut microbial community [69]. While the exact mechanisms remain to be fully elucidated, integrating olive oil into a Mediterranean dietary pattern may represent a valuable strategy for preventing and managing of hypertension and cardiovascular diseases.

### Fruits and vegetables

Among the fundamental components of the MedDiet are fruits and vegetables [76]. The consumption of fruits and vegetables not only promotes a dietary balance, but also plays a crucial role in reducing inflammatory processes, cancer prevention, oxidative stress, blood pressure and gut microbiota regulation [77, 78]. Furthermore, the American Heart Association (AHA) and the European Society of Cardiology (ESC) recommend the consumption of specific antioxidant phytochemicals and bioactive compounds present in fruits and vegetables to prevent certain cardiovascular diseases [79]. Notably, the intake of fruits such as oranges, red fruits, cocoa, among others, produces bioactive compounds such as polyphenols [80].

Polyphenols, compounds derived from vegetables, produce microbial modifications in the intestine, providing beneficial health effects at the microbiota level [81]. Studies suggest that polyphenol absorption is 5–10% of the total intake in the small intestine, and it is subsequently deposited in the large intestine. The microbiota converts polyphenols into low molecular weight metabolites and bioactive compounds. These products are then released into the bloodstream and are chemically different from their original structure. Furthermore, the microbiota can be modified by polyphenols promoting the abundance of beneficial bacteria [82].

Quercetin, a polyphenol found in apples, berries, and red fruits, has been associated with a decreased CVD risk [83]. For instance, in a systematic review and meta-Analysis of randomized controlled trials by Serban MC. et al. (2016), the results showed significant reductions blood pressure. For instance, systolic blood pressure had a weighted mean difference of − 3.04 mmHg, with 95% confidence interval of − 5.75 and − 0.33, and a *p-value* of 0.028. Similarly, diastolic blood pressure had a weighted mean difference of − 2.63 mmHg, with 95% confidence interval of − 3.26 and − 2.01, and a *p-value* of < 0.001 [84]. [NO_PRINTED_FORM] In addition, quercetin intake could modify the biodiversity of the gut microbiota by promoting the proliferation of beneficial bacteria such as genera *Bacteroides, Bifidobacterium*, and *Lactobacillus* promoting the homeostasis of the intestinal environment [85].

Furthermore, other studies mention that the consumption of plant foods containing phenolic compounds is associated with the increased abundance of some bacterial genera such as Bifidobacterium, and Schisandra chinensis. Moreover, some bioactive phenols, found in herbal teas, may have antimicrobial functions against *Helicobacter pylori, Escherichia coli*, among others [80].

Nut consumption has been associated with an increased abundance of beneficial bacteria in the gut microbiota, further promoting the production of short-chain fatty acids (acetate, butyrate, and propionate), which are beneficial for human health [86]. For example, pistachio components such as fatty acids and fibers promote the increase of microorganisms in fecal microbiota [87].

Moreover, in a study by Terzo S. et al. (2020), mice were fed a high-fat diet accompanied by pistachios. The authors found in the fecal samples of these mice that inflammation markers, such as cytokines TNF-α and IL-1β decreased, compared to mice fed a standard diet [88]. Notably, elevated levels of TNF-α and IL-1β promote inflammatory processes and abnormal immune responses, further contributing to hypertension [89, 90]. Therefore, pistachios may promote normal blood pressure by enhancing SCFA production and decreasing inflammatory cytokines [91]. Furthermore, in the same study, it was observed that various bacterial genera, such as *Parabacteroides, Dorea, Allobaculum, Turicibacter, Lactobacillus, and Anaeroplasma* significantly increased, while bacterial genera related to inflammation, such as *Oscillospira, Desulfovibrio, Coprobacillus, and Bilophila*, decreased [88].

Peanuts are another type of nut, and research has shown in animal models that their consumption can improve intestinal microbiota and ameliorate metabolic syndrome and its associated risk factors (obesity, hypertension, among others). However, further research is needed to elucidate the plausibility of extrapolating these results to humans [87].

Other essential components of the Mediterranean diet are legumes. The most consumed legumes in this diet are peas, beans, chickpeas, and lentils [92]. The consumption of these vegetables provides protein, starch, vitamins, and fiber, necessary elements of a daily diet [93]. Research has shown that a balanced diet based on legumes can reduce the risk of various diseases, including cancer, hypertension and cardiovascular diseases [92, 94]. Furthermore, the consumption of vegetables can modify microbial diversity, and an increase in *Bifidobacteria* and *Bacteroides* species, and other SCFA-producing bacteria, such as *Eubacterium rectale* and *Clostridium leptum*, has been described [95].

Thus, a diet rich in fruits and vegetables has beneficial effects on intestinal microbiota, which, in turn, may aid in blood pressure regulation in patients with hypertension [82]; however, only a few studies have shown a direct route of action of phenolic compounds on blood pressure [33, 96].

### Wine

In recent years, the association of gut microbiota dysbiosis with the emergence of non-transmissible diseases like CVD has been widely reported [97]. For instance, in a hypertensive Korean population sample, a lower gut microbiota diversity and depleted SCFA-producing bacteria (*Faecalibacterium, Blautia, Anaerostipes*) have been observed [98]. This low concentration of SCFA could be restored by acetate supplements and a high-fiber diet, which improves blood pressure in hypertensive mice [99]. It has been reported that the SCFA can decrease blood pressure through the regulation of renin secretion by the G-protein coupled receptor pathway [100].

Another study also showed diversity reduction and shifts in the microbial composition of Chinese hypertensive individuals [101]. Moreover, these Chinese hypertensive individuals had higher pathogenic bacteria distribution *(Klebsiella spp., Streptococcus spp., and Parabacteroides*) than healthy controls [101], which has been involved in microbial translocation, systemic circulation, and atherosclerosis progression [97]. Moreover, a Brazilian study found a microbiota dysbiosis with decreased butyrate producers (*Roseburia and Faecalibacterium*) and increased TNF-α and IL-6 cytokines production [102]. This evidence supports the role of gut microbiota and its metabolites in blood pressure modulation.

Gut dysbiosis may be caused by environmental factors such as diet [103, 104]. For instance, a microbiome enhancer diet (high fiber, minimally processed food) has been shown to increase microbiota diversity and SCFA production [105]. Recently, moderate alcohol consumption (0–40 g of pure alcohol per day) has been reported to improve the cardiovascular health of coronary artery disease individuals by reducing serum concentrations of sphingolipids and glycerophospholipids [106]. Moreover, moderate alcohol consumption has been associated with the depletion of pathogenic bacteria (*Bacteroides ovatus, Bacteroides coprocola, Enterococcus villorum*), and an increase in beneficial species (*Oscillibacter valericigenes, Paraprevotella clara*) [106].

The specific mechanisms of this bacterial modulation have not been established; however, it has been reported that polyphenols can alter the microbial membrane of specific bacterial groups in a dose-dependent manner [107, 108]. It has also been described that polyphenols can alter bacterial DNA synthesis, inhibit energy metabolism, interfere with quorum sensing, and disrupt biofilm generation. The ability of polyphenols to form complexes with proteins has been reported as the mechanism implicated in disrupting these processes [109, 110]. Moreover, the metabolites generated after the metabolism of polyphenols have also been linked with bacterial modulation, although this approach requires further studies due to limited evidence [111]. Therefore, these approaches could encourage more research to improve the understanding of the role of red wine compounds in the modulation of gut microbiota and their health benefits.

Red wine is a specific alcoholic drink that has demonstrated beneficial effect on cardiovascular health. It has been shown to improve antioxidant status [112, 113], gut microbiota [107, 114], lipid profile [115, 116], thrombosis risk [117], and inflammatory markers [118]. However, there is substantial controversy regarding the benefits of red wine consumption in human health, particularly due to the wide pathologies associated with alcohol abuse, such as hypertension, liver disease, and cancer [119]. Furthermore, interindividual differences, including genetics, lifestyle, and gut microbiota composition, could define the outcomes of alcohol exposure [120]. Therefore, these individual factors could influence human health following alcohol exposure.

Resveratrol, a polyphenolic compound found in red wine and grapes, can reduce blood pressure by inhibiting angiotensin II through the modulation of NF-κB signaling in murine models [121, 122]. Furthermore, it has been proposed as an anti-atherogenic factor [121, 122]. The effects of polyphenols in the modulation of intracellular signaling pathways could be associated with their role in the inhibition or stimulation of the phosphoinositide 3-kinase, Akt/protein kinase B, tyrosine kinases, protein kinase C, mitogen-activated protein kinase, AKT/mTOR, and adenosine monophosphate-activated protein kinase signaling cascades [123, 124]. Therefore, it is important to conduct studies assessing the effects of the metabolites produced by specific bacterial species to understand their role in the regulation of metabolic pathways and their correlation with human health [121] Moderate red wine consumption (250 mL per day, five days per week) also remodeled the gut microbiota in adult men. There was a predominance of *Parasutterella, Ruminococcaceae, Bacteroides species*, and *Prevotella*; moreover, a change in the serum metabolome associated with improved antioxidant effect was observed [125].

The effect of red wine on gut microbiota has shown that several beneficial bacteria (*Lachnospiraceae, Clostridales, Lactobacillus*, and *Ruminococcacceae*) are increased in pig animal models [126, 127]. Moreover, beneficial *Bifidobacterium* and *Prevotella* were increased in human gut microbiota after chronic red wine consumption, which was correlated with a lower concentration of liposaccharides in plasma [128]. The human gut microbiota diversity is also improved after red wine consumption, with an increased presence of the beneficial *Barnesiella* bacteria [129].

Therefore, red wine consumption has been correlated with increased gut microbiota diversity and abundance of various bacteria genera, such as *Faecalibacterium, Blautia, Anaerostipes*, *Bifidobacterium*, *Prevotella*, and *Barnesiella*. These bacteria may play a role in enhancing the metabolites levels in serum, including increasing HDL cholesterol, and decreasing sphingolipids, glycerophospholipids. These metabolic changes have been associated with improved cardiovascular health and could contribute to lowering blood pressure in hypertensive individuals. Nevertheless, the relationship between moderate alcohol consumption and human health is a topic of debate in the scientific community, and further research is needed to fully understand these associations. Therefore, while these findings are promising, it is important to remember that alcohol consumption should be moderate and part of an overall healthy lifestyle.

### Fish and lean proteins

Protein sources that are low in calories and saturated fat are known as lean proteins. A lean protein source is defined by the United States Department of Agriculture (USDA) as having less than 10 g of total fat (4.5 g or less of saturated fat) and fewer than 95 milligrams of cholesterol in a 100-gram serving [130].

Consuming more fat increases hypertension risk and causes intestinal dysbiosis [131]. According to Hsu et al. (2019), in hypertensive rats, a high-fat diet is linked to changes in the gut microbiota and suppression of nutrient-sensing signals. The study team looked at the effects of a high-fat diet on blood pressure in adult male rat offspring both during and after weaning. The study found that a high-fat diet throughout pregnancy and after weaning caused blood pressure to rise. Furthermore, this same diet increased the *Firmicutes*/*Bacteroidetes* ratio, the abundance of the genus *Akkermansia*, *Clostridium*, *Alkaliphilus* and the phylum *Verrucomicrobia*. Moreover, it also reduced the abundance of the genus *Lactobacillus*, *Parabacteroides* and *Ruminococcus* [132].

Similarly, Chen et al. (2023) conducted a systematic review, revealing that a high-fat diet can lead to gut microbiota dysbiosis by influencing the metabolism of bile acids, trimethylamine N-oxide (TMAO), and short-chain fatty acids (SCFAs). This dysbiosis involves alterations in the abundance of butyric acid-producing bacteria, opportunistic pathogens, *Lachnoclostridium*, unidentified Enterobacteriaceae, and SCFA-producing bacteria [133]. Furthermore, Sun et al. (2022) showed that intestinal microbial metabolites, including SCFA, TMAO, and endotoxins (lipopolysaccharides, LPS), have a substantial influence on hypertension by affecting undulatory vasomotion, renal function, neuronal activation, and inflammation [134].

Conversely, Wang et al. (2019) performed a study on 217 healthy young people, in which the authors analyzed three isocaloric diets, a low-fat diet (fat 20% of energy), a moderate-fat diet (fat 30% of energy), and a high-fat diet (fat 40% of energy). The research found that a low-fat diet raised the number of *Blautia* and *Faecalibacterium* as well as the α-diversity of the gut microbiota. On the other hand, a high-fat diet decreases the number of *Faecalibacterium* and increases the abundance of *Bacteroides* and *Alistipes* [135].

According to research by Wolters et al. (2019) microbiota diversity is inversely correlated with a high fat and saturated fatty acid consumption. Moreover, saturated fatty acids promote the enrichment of *Clostridium* and *Blautia* [136].

## MedDiet and DASH

Recent studies have highlighted the efficacy of dietary combinations in blood pressure regulation, such as MedDiet and Dietary Approaches to Stop Hypertension (DASH). The DASH diet is characterized by reduced saturated fat, total fat, and cholesterol intake, with high levels of fiber, protein, potassium, magnesium, and calcium sourced from fruits and vegetables [137]. Zhang et al. (2022) examined the impact of these dietary patterns on blood lipids across seven ethnic groups, revealing that MedDiet and DASH diet were negatively associated with blood lipids [138]. Additionally, several studies have elucidated the close relationship between lipid profile and hypertension [139–141]. Similarly, a study on the Iranian population compared adherence to the DASH diet and MedDiet with blood lipid levels, demonstrating that high adherence to the MedDiet was associated with lower odds of LDL/HDL, which could indirectly influence blood pressure regulation [142]. Another study found that adherence to the MedDiet, but not the DASH diet, was linked to a lower risk of all-cause mortality, particularly in individuals with diabetes, suggesting the potential effects on blood pressure regulation [143]. Furthermore, Filippou et al. (2023) studied the effects of MedDiet and DASH diet with salt restriction in adults with normal blood pressure and hypertension. Their findings were that MedDiet lowered systolic blood pressure, while combining the MedDiet with the DASH diet reduced blood pressure compared with salt restriction alone [144]. These findings suggest the potential of the MedDiet over other dietary approaches in enhancing cardiovascular health and managing blood pressure.

## Potential harmful effects of MedDiet components

The MedDiet and its components are linked to various health benefits. Nevertheless, the MedDiet also includes occasional consumption of red meat and processed foods. These food types have been associated with potentially harmful effects, including an increased risk of mortality, different types of cancer, and various chronic diseases [145–149]. Furthermore, red meat and processed foods may have repercussions on the gut microbiota. For instance, Kohnert et al. (2021) performed a randomized controlled trial to determine the effects of a 4-week intervention comparing a vegan diet with a meat-rich diet on the gut microbiota of healthy participants. The study did not find differences between alpha and beta diversity; however, it identified an increase in the *Faecalibacterium* and *Roseburia* genera, as well as a decrease in the *Coprococcus* genus [150]. Interestingly, the *Coprococcus* genus has been linked to normal blood pressure [151]. Conversely, a study by Yan. et al. (2017) found that hypertensive patients had a higher abundance of species from the *Roseburia* genus [152], whereas, for the *Faecalibacterium* genus, the results are conflicting; some studies have associated a higher abundance of the genus with hypertension, while others have correlated a lower relative abundance with hypertension [152–154].

Moreover, a systematic review conducted by Wang. et al. (2023) on the association between gut microbiota and meat consumption identified various key findings. Firstly, there is a limited number of randomized controlled trials investigating this association. Secondly, the results regarding the types of bacteria are inconsistent. Thirdly, there are inconsistencies in study designs. Lastly, the current evidence is insufficient. Therefore, further research is encouraged to fully understand the impact of consuming meat on gut microbiota and overall health [155].

## Future directions

Studies focusing on the MedDiet, gut microbiota composition, and blood pressure could explore various areas. For instance, examining the impact of this dietary pattern in different types of cells, including cells involved in blood pressure regulation processes such as red blood cells, endothelial cells, cardiomyocytes, smooth muscle cells, and cardiac fibroblasts. Furthermore, the integrative use of other -omics techniques could further improve the understanding of the molecular mechanisms associated with the interplay between MedDiet, microbiota, and blood pressure.

## Conclusions

The Mediterranean Diet, rich in whole grains, olive oil, fruits, vegetables, wine, fish, and lean proteins, has been associated with numerous health benefits. In the present article various components of the MedDiet are described along with their potential mechanisms for fostering a healthy microbiota and regulating blood pressure. For instance, whole grains are high in dietary fiber, which can promote a healthy gut microbiota and regulate blood pressure by producing SCFAs. Similarly, fruits and vegetables, not only provide dietary balance but also have potential regulatory effects on blood pressure and gut microbiota via polyphenols. Furthermore, the article presents the potential adverse effects of red meat and processed foods, which are occasionally included in the MedDiet, and their potential impact on human health. The article also identified gaps in the current knowledge and suggests different possible directions for future research in this field.

## Data Availability

All the data is presented in the manuscript.
